# RETRACTED: Richards et al. Protein Tyrosine Phosphatase Non-Receptor 11 (*PTPN11*/Shp2) as a Driver Oncogene and a Novel Therapeutic Target in Non-Small Cell Lung Cancer (NSCLC). *Int. J. Mol. Sci.* 2023, *24*, 10545

**DOI:** 10.3390/ijms27093976

**Published:** 2026-04-29

**Authors:** Cathy E. Richards, Yasir Y. Elamin, Aoife Carr, Kathy Gately, Shereen Rafee, Mattia Cremona, Emer Hanrahan, Robert Smyth, Daniel Ryan, Ross K. Morgan, Susan Kennedy, Lance Hudson, Joanna Fay, Kenneth O’Byrne, Bryan T. Hennessy, Sinead Toomey

**Affiliations:** 1 Medical Oncology Group, Department of Molecular Medicine, Royal College of Surgeons in Ireland, D09 YD60 Dublin, Ireland; yyelamin@mdanderson.org (Y.Y.E.); aoife.carr@hotmail.com (A.C.); mattiacre@gmail.com (M.C.); robertsmyth23@gmail.com (R.S.); danieljohnryan@beaumont.ie (D.R.); bryanhennessy74@gmail.com (B.T.H.); sineadtoomey@rcsi.ie (S.T.); 2 Department of Thoracic Head and Neck Medical Oncology, Division of Cancer Medicine, M.D. Anderson Cancer Centre, Houston, TX 77030, USA; 3 Thoracic Oncology Research Group, Trinity Translational Medicine Institute, Trinity College Dublin, St. James’s Hospital, D08 NHY1 Dublin, Ireland; kgately@stjames.ie (K.G.); shereen.rafee@gmail.com (S.R.); 4 Department of Medical Oncology, St. Vincent’s Hospital, D04 T6F4 Dublin, Ireland; emerohanrahan@gmail.com; 5 Department of Respiratory Medicine, Beaumont Hospital, D09 V2N0 Dublin, Ireland; rossmorgan@beaumont.ie; 6 Department of Pathology, St. Vincent’s Hospital, D04 T6F4 Dublin, Ireland; s.kennedy@st-vincents.ie; 7 Department of Surgery, Royal College of Surgeons in Ireland, D09 YD60 Dublin, Ireland; lhudson@rcsi.ie; 8 RCSI Biobank Service, Royal College of Surgeons in Ireland, D09 YD60 Dublin, Ireland; joannafay@rcsi.ie; 9 Princess Alexandra Hospital, Brisbane, QLD 4102, Australia; k.obyrne@qut.edu.au

*IJMS* is retracting the article titled “Protein Tyrosine Phosphatase Non-Receptor 11 (*PTPN11*/Shp2) as a Driver Oncogene and a Novel Therapeutic Target in Non-Small Cell Lung Cancer (NSCLC)” [1], cited above.

Following publication, concerns were brought to the attention of the Editorial Office regarding the integrity of a figure presented in this publication [1].

In accordance with the journal’s standard procedures, the Editorial Office and Editorial Board conducted an investigation that identified indications of inappropriate editing in Figure 4 and duplication between Figure 3D in [1] and Figure 3D in [2], previously published by a different author group. Although the authors cooperated with the investigation, the explanations and supporting materials provided were not deemed sufficient by the Editorial Board to resolve the identified concerns. As a consequence, the Editorial Board has lost confidence in the reliability of the reported findings and has decided to retract this publication in accordance with MDPI’s retraction policy.

This retraction was approved by the Editor-in-Chief of *IJMS*.

The authors have been informed of this retraction and have indicated their agreement with this decision.
